# Logical Framework Approach a Platform for Integrating the Mental Health and Nutritional Care for Controlling the Covid-19 Pandemic

**DOI:** 10.18502/ijps.v15i4.4299

**Published:** 2020-10

**Authors:** Seyed-Ali Mostafavi, Parviz Molavi, Fatemeh Mohammadi

**Affiliations:** 1 Psychiatry and Psychology Research Center, Roozbeh Hospital, Tehran University of Medical Sciences, Tehran, Iran.; 2 Department of Psychiatry, Fatemi Hospital, School of Medicine, Ardabil University of Medical Sciences, Ardabil, Iran.; 3 School of Social Sciences, Allameh Tabataba’i University, Tehran, Iran.

**Keywords:** *Coronavirus*, *COVID-19*, *Logical Framework Approach*, *Mental Health*, *Nutrition*, *Pandemics*

## Abstract

Coronavirus infection (COVID-19) is spreading rapidly in the world beyond the health care capacity. The World Health Organization (WHO) has announced an emergency state that needs quick and effective actions. In the lack of specific medicine and vaccine, integration of mental health and nutritional care in the platform of a powerful managerial technique named the “Logical Framework Approach” (LFA) could be helpful for successful control of this pandemic. The strengths of the LFA for coronavirus management program are stakeholders’ involvement, integrative teamwork in research and medical procedures, as well as Inter-sectoral cooperation. The related organizations like WHO and ministries of health of every country could easily adopt this approach and act more efficiently to manage this pandemic.

Coronavirus disease 2019 (COVID-19) pandemic has raised serious concerns about health care capacity worldwide. The spread of COVID-19 was so fast that nearly all countries are engaged by its infection (1). Although, many laboratories, research institutes, and universities worldwide are working to discover specific treatments and vaccines for COVID-19 infection, it is spreading among the people. 

In addition to health and medical issues, coronavirus infection and its related death have many psychological consequences for the patients and their families (2). Additionally, mental health and nutritional status of patients may interact with treatment of patients, and may take role in prevention of the infections and management of the pandemic (3). However, mental health and nutritional status of patients with COVID-19 infection are overlooked and urgent research and action in these fields are needed. 

Furthermore, coronavirus infection has induced a great economic burden to the governments (4). Hence, countries and companies start to spend money and act against coronavirus infection. However, the coronavirus prevention programs used by some countries have not been effective so far. One reason for this failure could be lack of the systematic view in some policy-makers and not engaging all stakeholders and related expertise. Hence due to importance of nutritional care and mental health in prevention and treatment of the disease, we aimed to write a narrative review on the “Logical Framework Approach” (LFA) as an overlooked platform for integrating mental health and nutritional care into the management programs for covid-19 infection. In this emergency condition of pandemic, we believe that integration of nutritional and mental care to the regular medical care of coronavirus infected patients would be cooperative.

We choose to review the LFA because it is a powerful management method with capacity of analyzing the hierarchy of problems and integrating the solutions to achieve the goal. Hence, we decided to write this narrative review to help researchers and policy-makers in their programs for controlling the COVID-19 pandemic.

**Integration of mental health to the medical procedures for coronavirus treatment**

We are at beginnings steps of the new coronavirus pandemic. However, based on the literature, similar pandemics of SARS, MERS, and Ebola, the infection has serious psychological consequences for the patients and their family, in addition to medical problems (5, 6). Several studies have reported the short-term and chronic psychological consequences of previous coronaviruses related with sever anxiety, depression, distress and post-traumatic stress disorders (5-7). Furthermore, anxiety in critically ill patients will provoke the pathways of inflammation (8). On the other hand, decreasing anxiety and depression in patients may enhance the immune system and similarly may be effective in treatment of coronavirus patients (9). Hence, integration of mental health care to the regular medical treatments could be effective for the new coronavirus patients. 

**Integration of nutritional care to the medical procedures for coronavirus treatment**

Nutritional and dietary factors may help the coronavirus infected patients in two mechanisms. One, by decreasing the inflammation pathways in the respiratory system, and second by enhancing the immune system (10, 11). Knowing the immunosuppressant effect of protein-energy malnutrition in critically ill patients, the role of nutritional support for coronavirus patients in ICU wards is highlighted. Previous studies suggest that dietary anti-inflammatory factors and antioxidant nutrients such as vitamin C, vitamin D, vitamin E, omega-3, beta-carotene, and selenium and zinc may enhance the immune and respiratory systems (12). Accordingly, integration of nutritional interventions could be effective in treatment of the new coronavirus infection.

**LFA could be helpful in Coronavirus management programs**

The LFA is a powerful project management tool with pervasive use in many subject areas including medical and health projects (13). LFA is a managerial approach for implementation, achievement, and monitoring of any program including projects in health care systems (14). While many companies and agencies are now using this approach in managing their projects, its application is overlooked in health care programs. The WHO, governments, universities and institutes could easily adopt this approach and act more efficiently to slow down the spread of coronavirus. Therefore, we aim to introduce this management approach here. 

The LFA for coronavirus pandemic management may include five key analytical and logical elements to help integrative actions (15-18): 

1.   Problem analysis: Is the process of finding the core problem (rapidly spread of coronavirus in the world) causes and effects related to coronavirus infection. Furthermore, priorities are determined. This technique is similar to the problem tree analysis (16). 

2.   Stakeholder analysis: Is the process of identifying any individual, group, organization or system that could help in coronavirus control project or could be suffering from the coronavirus control project. The brain storming technique can be used to identify the stakeholders. The project manager also can map the interest & power matrix of stakeholders separately outside the LFA matrix (17). 

3.   Analysis of objectives: The main objective should be determined according to the core problem. Then the main objective should be broken down to the smaller and more manageable objectives in order to achieve the goal. The hierarchy of objective could be plotted like an objective tree (16). 

4.   Analysis of strategies: Identifying and comparing different solutions for coronavirus issue in order to control the pandemic (18). These strategies in coronavirus crisis may include community-based education, Regulations imposed by governments, encourage quarantine if necessary, Vast educational programs, TV programs & advertisements, Early detection and isolation of positive cases and People they've contacted, coronavirus vaccine development program, coronavirus medicine development program, Promoting mental health, nutritional status and complementary medicine (19, 20). 

5.   Analysis of the other factors: The process of identifying any systems, ethnics, countries, societies, religious, cultures and the other parameters as social, political, economical, legal, environmental and technological, which have the effects on the pandemic; and analyzing the symptoms to find the causes, and decreasing the problems and then control it (21). 

The above analytical approach is used to set up a management framework named Logical Framework Matrix. This Logframe summarizes the essentials of project, hierarchy of objectives and activities and assumptions (vertical logic; columns 1 and 4 in table 1), and how the project's development, outputs and outcomes will be monitored and assessed (horizontal logic; columns 2 and 3 of table 1). An example of a Logical Framework Matrix for management programs of coronavirus is presented in table 1. In this matrix, in addition to task priority, a milestone is being set (16, 21). Moreover, responsibilities, cost and budget are determined carefully for each task (table 2). The executive guarantor of the milestone activities for coronavirus control program is the portfolio management office in the WHO which is delegated to the Ministry of Health in each country.

The strengths of the logical framework approach for coronavirus management program are stakeholders’ involvement, integrative team work in research and medical procedures, as well as Inter-sectoral cooperation in all parts of society. Moreover, in the logframe responsibilities, cost and budget are determined carefully for each task, which leads to an arranged process to achieve the goals. In the absence of specific medicine and vaccine for coronavirus infection, adjunction of the regular medical care for the patients with complementary medicine, mental health care and nutritional care should be addressed (22, 23). 

Figure 1 shows the dynamic association of COVID-19 managerial process with Political, Economic, Social, Technological, Legal and Environmental (PESTEL) factors. In these critical days, unfortunately, political and economic situations may negatively influence on coronavirus management programs in some countries. In the case of Iran which are under the most severe and cruel political and economic sanctions which has affected medical affairs are more vulnerable to develop epidemic in spite of excellent health care professionals (24). Hence, stopping the political sanctions is essential to win the war against viruses.

**Table 1:** Example of a Logical Framework Matrix for Coronavirus Management Programs

**Project outline**	**Measurable indices**					**Method of affirmation**		**assumptions**
GOAL: Reducing the incidence of coronavirus and associated death	%Contribution of coronavirus to total death rate					Reduction of the percent which coronavirus contributes to total death rate		(Goal to super goal): successful Coronavirus portfolio management
PURPOSE: 1- Provide virus free environment for all people 2- Informing all people in the society about coronavirus 3- Equip sufficient hospital beds for coronavirus infected patients 4- Inventing new medicine for treating people affected by coronavirus 5- Inventing new coronavirus vaccine	- % of people access to virus free environment - % of people informed about coronavirus ways of transition and protection - % of empty beds devoted to coronavirus infected patients - % of patients treated by new medicines according to clinical trial studies - % of people who developed immunity against coronavirus					Incidence of coronavirus infection as will report by ministry of health officials Rates of recovering from coronavirus infection as will report by ministry of health officials		(Purpose to goal) Successful enrolment of all stakeholders and their contribution to reach the objectives and goals
OUTPUTS: 1- Promoting the knowledge, attitude and practice of society members toward coronavirus infection 2 -Breaking the virus transmission chain 3- Finding coronavirus vaccine and medicine 4- Efficient prevention and treatment of coronavirus infection	%of people act according to the coronavirus prevention guidelines % of new cases or incidence of coronavirus infection % of people immunized by new coronavirus vaccine. % of people treated by new coronavirus medicines.					Total hours in TV programs devoted to healthy habits to prevent coronavirus KAP questionnaires Clinical trials of coronvirus vaccine and medicines		(Outputs to purpose) Well management process of activities and tasks
ACTIVITIES:								Activity to output) Well satisfied program requirements (such as PESTEL* analysis and SWOT analysis)
	INPUTS & RESOURCES:							
1- Community-based	No. of health care	Month 1	Month 2	Month 3	Month 4	Month 5		
Education	professionals that							
2- Regulations imposed	focus on							
by governments	community							
3- Encourage	education and							
quarantine if	treatment							
necessary	No. of hospital beds							
4- Vast educational	and equipments							
Programs	devoted to							
5- TV programs &	coronavirus infection							
Advertisements	No. of scientists and							
6- Early detection and	researchers that							
isolation of positive	focus on developing							
cases and People	vaccines and							
they've contacted	medicines							
7- Coronavirus vaccine	Supplies expend in							
development	development of							
program	vaccine							
8- Coronavirus	Supplies expend in							
medicine program	development of new							
9- Promoting mental	medicines							
health, nutritional	Legislations which							
status and	support this program							
complementary	No. of agencies and							
medicine	organizations dealing							
	with coronavirus issue							
	Funding and other							
	resources that							
	support this program							
	Total Cost							
	No. of health care							
	professionals that							
	focus on community							
	education and							
	treatment							

* PESTEL stands for Political, Economic, Social, Technological, Legal and Environmental,

+ SWOT stands for Strengths, Weaknesses, Opportunities and Threats

**Table 2:** Milestone Activities for Coronavirus Control Program (Detailed list of Activities and Resources Allocation to each Task Could be Filled in This Timeframe)

**Activity**	**Sub-** **Activity ** **(Task )**	**Duration**	**Year 2020**					**Responsibility** ** (Name of ** **Person / ** **Organization)**	**Requirements**	**Materials & ** **Equipments**	**Cost**	**Budget**
			**April**	**May**	**June**	**July**	**August**					
				——›	——›							
1.					——›	—›						
2.						—›	——›					
3.												
4. etc												

**Figure 1:**
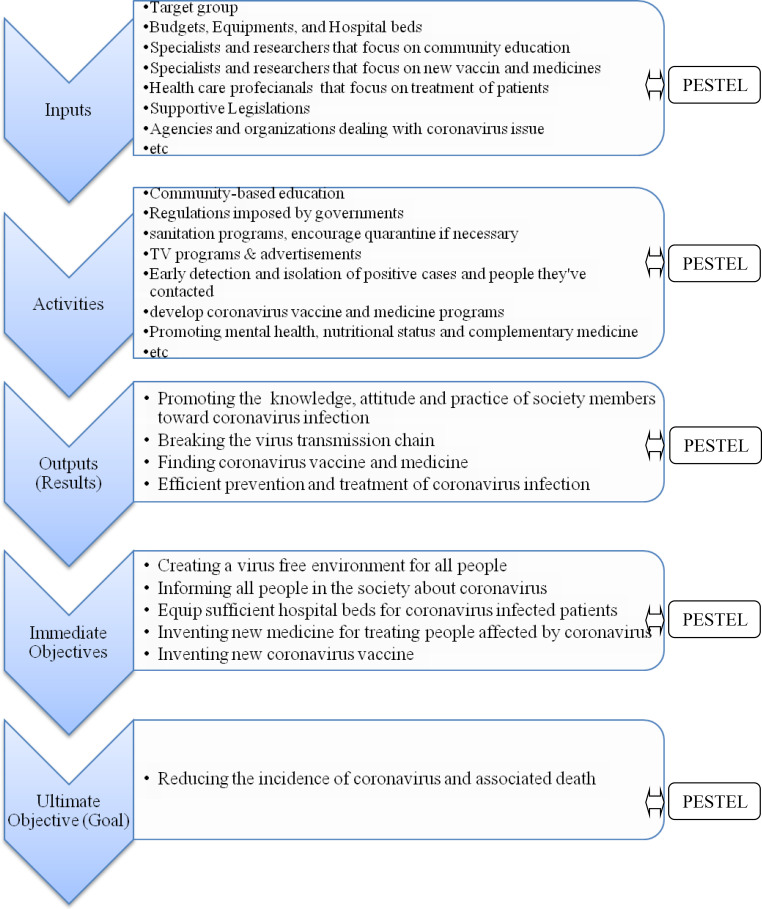
COVID-19 Control Process; ‹—› indicates the Dynamic Association of COVID-19 Managerial Process with Political, Economic, Social, Technological, Legal and Environmental (PESTEL) Factors

## Limitation

We are at the beginnings steps of the new coronavirus pandemic. Hence, lack of literature related to coronavirus is obvious. However, using the experience of previous outbreaks such as SARS, MERS and Ebola infections could be helpful.

## Conclusion

Currently weakness in coronavirus control programs is obvious. In the absence and also in presence of specific medicine for COVID-19 infection, mental health and nutritional interventions could be cooperative. LFA is a powerful management instrument that could be used by policy-makers for integration of the mental health and nutritional care to the prevention and treatment protocols of coronavirus infection. The key points of LFA for coronavirus control programs are integrative and teamwork approach, involvement of all stakeholders, government and people, and powerful portfolio leadership.
